# Tailoring implementation interventions of different order in infection prevention and control: A cascadic logic model (IPC-CASCADE)

**DOI:** 10.3389/frhs.2022.960854

**Published:** 2023-01-16

**Authors:** Thomas von Lengerke, Ivonne Tomsic, Karolin M. E. Krosta, Ella Ebadi, Valentine Keil, Frederike Buchta, J. Katrin Luz, Tiffany Schaumburg, Susanne Kolbe-Busch, Iris F. Chaberny

**Affiliations:** ^1^ Department of Medical Psychology, Hannover Medical School, Center for Public Health and Health Care, Hannover, Germany; ^2^ Institute for Medical Microbiology and Hospital Epidemiology, Hannover Medical School, Center for Laboratory Medicine, Hannover, Germany; ^3^ Institute of Hygiene, Hospital Epidemiology and Environmental Medicine, Leipzig University Hospital, Interdisciplinary Center for Infectious Medicine, Leipzig, Germany

**Keywords:** infection prevention and control, healthcare-associated infections, compliance, implementations interventions, tailoring, empowerment, team science, psychology

## Abstract

Implementation interventions in infection prevention and control (IPC) differ by recipients. The two target groups are healthcare workers directly involved in patient care (“frontline”) and IPC professionals as proxy agents, that is, implementation support practitioners. While both types of implementation interventions aim to promote compliance with clinical interventions to prevent healthcare-associated infections (HAI), their tailoring may be vastly different, for example, due to different behavioural outcomes. Additionally, IPC teams, as recipients of empowering tailored interventions, are under-researched. To overcome this gap and improve conceptual clarity, we proposed a cascadic logic model for tailored IPC interventions (IPC-CASCADE). In the model, we distinguished between interventions by IPC professionals targeting clinicians and those targeting IPC professionals (first- and second-order implementation interventions, respectively). Tailoring implies selecting behaviour change techniques matched to prospectively-assessed determinants of either clinician compliance (in first-order interventions) or interventions by IPC professionals for frontline workers (in second-order interventions). This interventional cascade is embedded in the prevailing healthcare system. IPC-CASCADE is horizontally structured over time and vertically structured by hierarchy or leadership roles. IPC-CASCADE aims to highlight the potential of increasing the impact of tailored interventions *by* IPC professionals for clinicians (to improve their compliance) *via* tailored interventions *for* IPC professionals (to improve their work as proxy agents). It underlines the links that IPC professionals define between macro contexts (healthcare and hospitals) and frontline workers in HAI prevention. It is specific, i.e., “tailored” to IPC, and expected to assist implementation science to better conceptualise tailoring.

## Introduction

Infection prevention and control (IPC) can be defined as the evidence-based practice of preventing and controlling healthcare-associated infections (HAI); its key purpose is to protect patients and healthcare workers from HAI as an unwanted side effect of healthcare. Clinical interventions, such as hand hygiene, surface disinfection, sterile handling, and wound management, can prevent HAI directly, at least if they are implemented compliantly, that is, according to guidelines and following the best available evidence. However, scientific evidence is usually insufficient for promoting compliance with HAI-preventive measures because ‘…change is achieved by individuals within the organisations, who need to align new interventions and practices with their own education, beliefs, and perceptions, and the context in which they work’ (1), p. 6]. This has led IPC to increasingly draw on implementation science (1–6). In particular, the distinction between implementation and clinical intervention has been utilized (7). While an implementation intervention denotes a ‘…method or technique designed to enhance adoption of a “clinical” intervention…’ (8), p. 218], a clinical intervention refers to a ‘…clinical/therapeutic practice…or delivery system/organizational arrangement…or health promotion activity being tested or implemented to improve health care outcomes’ [ibid]. This facilitates the differentiation between HAI-preventive interventions with regard to immediate targets. For example, educational meetings on hand hygiene usually aim to improve healthcare workers' knowledge and motivation regarding hand hygiene as a clinical intervention. Here, the targets are healthcare workers directly involved in patient care (i.e., frontline providers), or more specifically, individual determinants of their behaviour (e.g., motivation). Similarly, providing disinfectant dispensers at optimal points-of-care represents another example of an implementation intervention directed at frontline providers, but targeting an environmental determinant of behaviour. In contrast, clinical interventions target processes more proximal to (preventing) HAI, for example, pathogen load on frontline providers' hands and pathogen transmission.

The implementation interventions considered thus far are usually organised and delivered to frontline providers by IPC professionals (hospital epidemiologists, IPC nurses, and hygiene engineers), and other stakeholders, such as hospital pharmacists or service engineers. Simultaneously, there are implementation interventions not being delivered *by* but targeted *towards* IPC professionals as proxy agents, i.e., implementation support practitioners (9). Usually, these aim to teach implementation skills to IPC professionals (1) and may be provided as part of in-house or external continuing education programs. Moreover, structural interventions may also fall into this category; for example, compliance with personnel and organizational requirements for HAI prevention, whereby hospital management directly targets IPC professionals and their work environment.

A potentially tricky aspect of this distinction is that both implementation interventions aim at the sustained adoption of clinical interventions, and eventually, HAI prevention and control. Thus, although their ultimate aims are very much the same, they differ in features which are highly relevant if one wants to tailor these interventions. Most importantly, they usually address very different behavioural outcomes, such as how to effectively disinfect one's hands (interventions addressing frontline providers) and how to effectively provide feedback on hand hygiene (interventions addressing IPC professionals). These behavioural outcomes imply different compliance requirements. Among other things, this is relevant for tailoring implementation interventions, i.e., the development of ‘…strategies to improve professional practice that are planned, taking account of prospectively identified determinants of practice’ (10), p. 5]. In general, the rationale for tailoring is similar to that of individualised medicine: tailored interventions are developed based on typical, empirically assessed attributes of their recipients that are relevant to an outcome such as hand hygiene compliance. This aims to improve its effects through better interventions-recipients fits. Currently, the scope of tailoring goes beyond its original application of promoting healthy behaviours in patients (11), and is also applied to healthcare workers’ professional behaviour (10). In the IPC context, the different behavioural outcomes (that are dependent on interventions' recipients) imply that both the determinants of practice (including contextual variables) and the selection and application of implementation strategies are often quite different. Both these differences and the tendency in IPC research so far to focus on either clinical or implementation interventions addressing frontline providers [see (7, 12, 13) for surgical site infections (SSI)] make it essential to distinguish between these two implementation interventions. In addition, the fact that even the basic distinction between implementation and clinical interventions (8) is neglected in many evidence-based guidelines [e.g., in German guidelines and recommendations on hand hygiene (14, 15)] suggests that conceptual confusion may be more than a potential liability.

Thus, to provide a more conceptual structure for the complexity of the interventional sequence inherent in clinical and different implementation interventions, we proposed a cascadic logic model for IPC interventions to prevent HAI. In doing so, we integrated the concept of tailoring as the selection of behaviour change techniques (BCT) (16–18) matched to empirically identified mechanisms of action, that is, determinants of behaviour, and highlighted differences in how tailoring may have to be approached as dependent on different intervention providers and recipients.

## Model development

**Figure 1:**
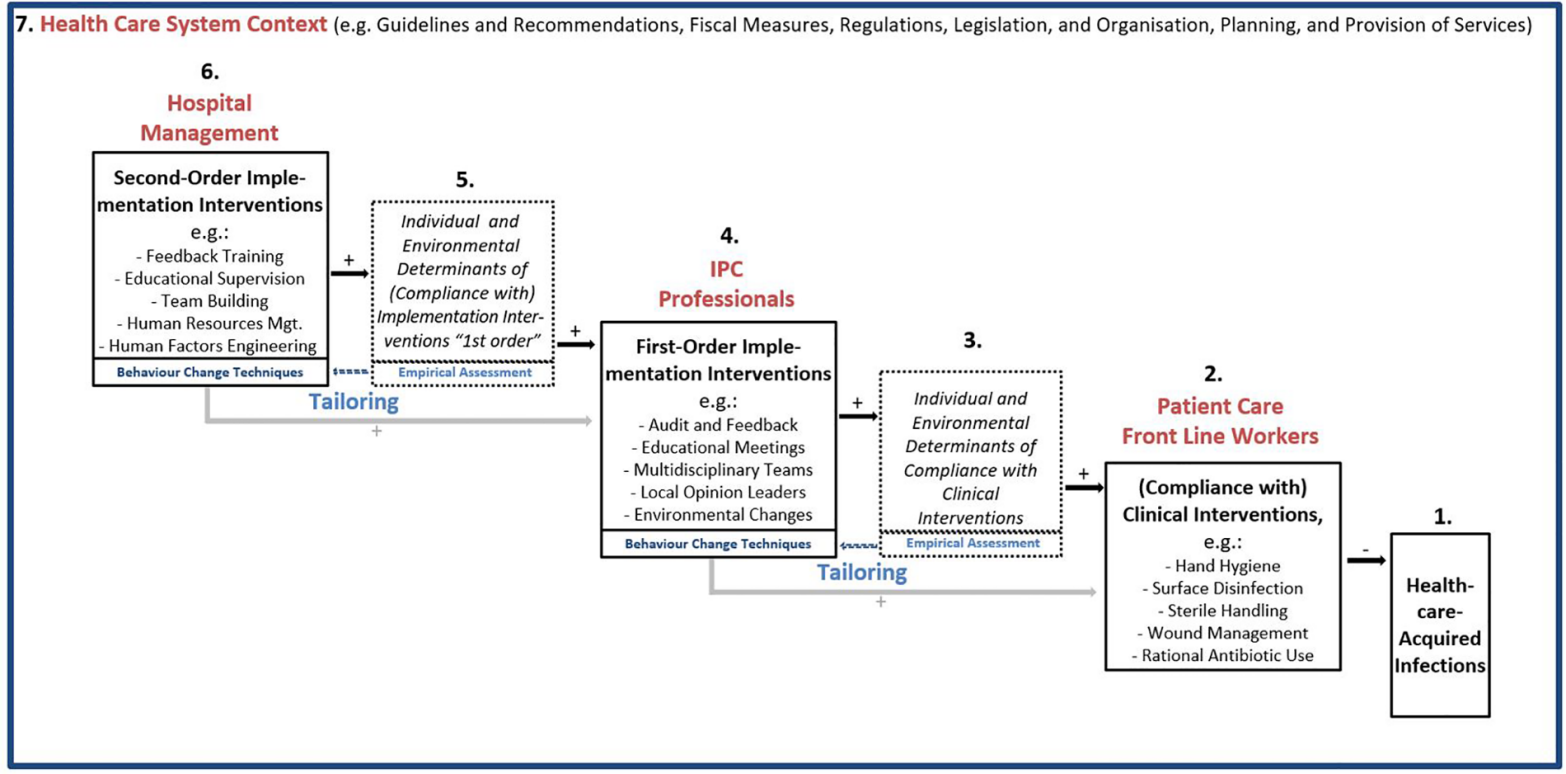
A cascadic logic model of infection prevention and control implementation and clinical interventions to prevent healthcare-acquired infections (IPC-CASCADE)^$^. ^$^ Feedback loops (e.g., from infections and compliance to second-order implementation interventions) and direct paths from hospital management to patient care frontline worker level omitted for clarity of presentation.

The development of the model consisted of two phases. In the first phase, a logic model was developed to distinguish between implementation and clinical interventions to prevent SSI (7); Figure 1]. Its trigger was a classification in the respective guidelines by the Association of the Scientific Medical Societies in Germany (14), in which preventive measures were grouped into four categories: pre-operative, intra-operative, post-operative, and interventions relevant to all expositional periods. While measures in the first three categories consistently represented clinical interventions (e.g., hair cutting or removal, antibiotic prophylaxis, and monitoring drainages), the last category included hand hygiene, surveillance, checklists, compliance audits and monitoring, and education and training. Clearly, the latter four items belong to a different level of intervention than hand hygiene (and other measures), because they aim to increase compliance with these rather than to prevent infections directly. These measures usually prevent infections indirectly, by targeting individual and environmental compliance determinants.

In the second phase, it became increasingly clear that there are variations in the content, quality, and impact of interventions such as checklists, and education and training, which are due to differences in the motivation, capabilities, and opportunities of the IPC professionals who deliver them (19–23). In other words, implementation support practitioners may require support themselves. As noted, their tasks may be quite different from those of frontline providers (e.g., educating about hand hygiene vs. hand hygiene), and because of a tendency to focus on interventions addressing frontline providers (7, 12,13), a model that allows distinguishing between different implementation interventions in IPC along the lines of different addressees was sought. This was deemed worthwhile for at least two reasons: first, for providing a conceptual basis for tailoring *via* selection of BCTs on multiple levels in the context of IPC; and second, for contributing to IPC's openness regarding perspectives from organisational behaviour as ‘…a field of study that investigates the impact individuals, groups, and structure have on behavior within organizations for the purpose of applying such knowledge toward improving an organization's effectiveness’ (24), p. 29f.].

## Interventions to promote compliance to prevent HAI: the logic model IPC-CASCADE

Figure 1 shows the proposed cascadic logic model IPC-CASCADE. On the lower right-hand side, the HAI is specified as a nosological entity to be prevented (1). Most proximately, evidence-based clinical interventions are directly effective in preventing HAI within the interventional sequence covered by the model (2). They are usually implemented by healthcare workers who are directly involved in patient care. Rational antibiotic use is a special case in this context, as it prevents antimicrobial resistance rather than HAI.

Moving upward to the left, there are several determinants of compliance with clinical interventions (3), which may differ depending on the intervention(s). In accordance with the psychological axiom that behaviour is always a function of the person and their environment(s) (25), individual and environmental determinants are distinguished. The most general encompassing models to systematise compliance determinants are the Capabilities|Opportunities|Motivation–Behaviour Model (COM-B) (26,27) and the Theoretical Domains Framework (28,29), which comprises a comprehensive list of constructs (19).

When tailoring interventions aimed at directly increasing compliance with clinical interventions (4), one would, by definition (10), empirically assess their specific determinants and match strategies to the identified determinants. This implies the selection of BCT (16–18), which is an ‘…observable, replicable, and *irreducible* component of an intervention designed to alter or redirect causal processes that regulate behavior… (16), p. 82, our emphasis]. Additionally, a BCT as ‘…an “active ingredient” … can be used alone or in combination and in a variety of formats’ [ibid.]. This implies that BCT may be a component of different implementation interventions that IPC professionals use to increase frontline workers’ compliance, such as audit, feedback, and educational meetings (to, for example, improve knowledge, motivation, and skills as individual factors) and environmental changes (e.g., providing alcoholic hand-rub dispensers at optimal points-of-care). As part of the tailoring process, their selection may be complemented by implementation strategies which do not directly target behaviour change (such as assessing for readiness and identifying barriers and enablers, and promoting adaptability, as suggested by a recent comparison of the Expert Recommendations for Implementing Change compilation and the BCT taxonomy (30). Regardless of the case, due to their positioning in IPC-CASCADE, we chose to term these *first-order implementation interventions* (those *by* IPC professionals addressing colleagues directly involved in patient care).

Going beyond earlier models [e.g. (7),], IPC-CASCADE duplicates this logic to describe the impact of *second-order implementation interventions* (6) which target IPC professionals to support them in their efforts to promote clinicians' compliance (i.e., first-order implementation interventions) and eventually prevent HAI. Similar to first-order interventions, Figure 1 provides examples, the potential effectiveness of which is in *Discussion*. Again, the *direct* impact of second-order implementation interventions is not on behaviour (i.e., conduct of first-order implementation interventions), but on individual and environmental determinants relevant to the work of the recipients (5), in this case, IPC professionals. For instance, the educational competencies of IPC professionals may be enhanced by educational supervision, and the identification of optimal points-of-care for hand rub dispensers can be advanced by human factors engineering. Additionally, outcomes, such as ensuring that staff delivering interventions maintain a minimum level of satisfaction and well-being during delivery, may be pursued. Once again, tailoring is pursued by empirically assessing the mechanisms of action, that is, the different determinants of the preventive work of IPC professionals, and then selecting the appropriate BCT.

This cascade of interventions is embedded in the context of the healthcare system in which they occur, including its guidelines, recommendations, fiscal structures, regulations, legislation, and organisation of services (7). For example, in Germany, there are recommendations by the Commission for Hospital Hygiene and Infection Prevention at the Robert Koch Institute regarding personnel and organizational requirements for HAI prevention (31) and the capacity for support of hospitals and other medical facilities by hospital epidemiologists (32). On this basis, it has become possible to analyse and at least approximately evaluate the fulfilment of minimal IPC staffing levels in different healthcare institutions (33). Generally, both types of implementation interventions are affected by, and need to adapt to, contextual conditions.

Horizontally, the model is structured implicitly over time. As shown in Figure 1, feedback loops from right to left are graphically omitted for ease of presentation. For instance, an increase in HAI observed by hospital surveillance may have repercussions on first-order implementation interventions, in that possible reasons for the increase may be highlighted and addressed. Additionally, feedback by clinicians regarding first-order implementation interventions to IPC professionals or hospital management may lead to investments by the management in building capacities regarding IPC professionals' skills to provide feedback, cope with resistance, and manage change, and more generally inform continuous adjustments of BCT as part of implementation support activities. Moreover, hospital management might affect the determinants of direct relevance to frontline staff, and thus “bypass” IPC professionals. This direct path is also omitted to ensure parsimony of the presentation in Figure 1. Generally, the time dimension is ideal-typical in that the logic model describes a multistep pathway from implementation to HAI prevention.

The vertical dimension (hierarchies and leadership roles) is also implicit in the model. This holds for hierarchies which exist within levels, for example, within IPC teams. This design, graphically represented from the top left to the bottom right, prompted the term “cascadic”. The vertical dimension allows the model to investigate the role of leadership when interprofessional teams at different levels are employed to reduce HAI (34). This includes IPC teams as proxy agents in terms of implementation support practitioners.

Finally, a general point must be considered: the model focuses on tailoring interventions which aim to optimise IPC professional behaviour and, eventually, make clinician *compliance* deliberate; if the ultimate goal is HAI prevention, there is no other path than following evidence-based guidelines in our view, which implies compliance. Of course, tailoring may target a much broader range of (implementation) goals to which the cascadic logic of the model may also be applied.

## Discussion

The most recent Cochrane review on tailored interventions targeting professional practice identified 32 studies and showed that these interventions had 1.56 times higher odds of leading to the desired outcomes than non-tailored interventions (10). Notably, however, all studies targeted clinical interventions such as antibiotic prescription in the community and influenza vaccination, to highlight those with direct IPC relevance. Thus, in terms of IPC-CASCADE, they scrutinised tailored *first-order* implementation interventions. Similarly, since the novel coronavirus disease (COVID-19) pandemic and its potential impediments to IPC, barriers and facilitators to compliance have been studied more thoroughly for healthcare workers directly involved in patient care than for IPC teams, as indicated by the only relevant Cochrane review since 2020 (35). The IPC-CASCADE model aims to address this gap and sensitise the potential of increasing the effects of tailoring IPC practice *via* first-order implementation interventions improved by tailored *second-order* implementation interventions. In other words, IPC teams, not only as providers but also as recipients of HAI-preventive interventions, are under-researched, and their empowerment is a blind spot in IPC.

Before further discussion of the evidence for the potential merits of the model, its limitations should be noted. First, as noted, it is deliberately applied to HAI-preventive compliance and thus restricted to this realm. Second, it does not claim to be a stringently multilevel model, which would imply that it is structured by a distinct individual level and at least one supra-individual level of analysis [i.e., following the basic behavioural epidemiology model (36)]. Instead, it is deliberately restricted at this point of its development to professional behaviours of different agents in the hospitals within an organisational system. Prospectively, it is planned to be further developed into a model not only of compliance of individuals, but compliance *rates* as well. Lastly, the model only implicitly addresses the challenge of tailoring by selecting BCTs based on a systematic linkage to mechanisms of action as “…theoretical constructs that represent the processes through which a BCT affects behaviour processes or events” [17, p. 694].

Bearing these limitations in mind, one study which aimed to provide empirical evidence for effective IPC by addressing IPC professionals is the Wound Infections and Antibiotics Use in Surgery (WACH) trial (German Clinical Trials Register-ID: DRKS00015502) (20). Drawing from conceptual analyses of IPC teams (37) and team science (38), it extends the approach of a preceding trial (21, 22). Specifically, while first-order implementation interventions to improve hand hygiene compliance in intensive care units of a tertiary care university hospital were the focus of that trial, WACH analysed COM-B-based tailoring of second-order implementation interventions to improve compliance with SSI prevention in surgical departments of non-university hospitals. Tailored interventions took the form of (1) a written report based on baseline assessments submitted to the IPC team, and (2) a multidisciplinary workshop with the IPC team and other IPC stakeholders in the study hospitals, with feedback training, educational supervision, and team building. Implementation strategies used in this context included organizational culture, audit and feedback, continuous quality, educational meetings or materials, reminders, interprofessional education, and local opinion leaders [labels are from the Effective Practice and Organisation of Care Taxonomy (39)]. Preliminary results show that, compared to usual practice, a compliance increase was observed after tailoring for 100% of clinical interventions included in the study (usual practice = 57%) (20, 23). Additionally, there was an increase only after tailoring in the proportion of operative procedures in which at least 80% of the pre- and intra-operative measures scrutinised were implemented (an effect further amplified after the onset of the COVID-19 pandemic) (20, 23). Although the final publication of its results is still pending, it can be tentatively inferred that tailored second-order implementation interventions hold promise for IPC.

More generally, IPC-CASCADE highlights IPC teams and the links the teams define between macro contexts (healthcare and hospitals) on one hand, and frontline healthcare workers and HAI on the other. It is not redundant to the research pipeline model (8), which distinguishes between efficacy, effectiveness, and implementation research, as both types of implementation interventions can be studied in terms of their efficacy, effectiveness, and implementation. In contrast, a stronger resemblance pertains to the model of multilevel behaviours in a hospital context (27), which specifies mechanisms of actions for behaviours of both frontline workers, and mid-level and senior managers. However, this model concentrates on hierarchies and does not specifically address IPC professionals or teams. In this sense, IPC-CASCADE is more specific and potentially more “tailored” to IPC.

Regarding potential implications for research and practice, IPC-CASCADE's potential merits are those of logic models in general: ‘… to represent the underlying theory of especially complex interventions in simple, diagrammatical form’, ‘… to help evaluators develop understanding of exactly how interventions produce outcomes,’ ‘… to organise empirical data and specify process and outcome measures for the purposes of evaluation”, “… and/or to provide a talking point for stakeholders to forge consensus on the need for change and how to go about it…’ (40), p. 2]. For hospitals and IPC teams, the model can be used to visualise the needs of IPC in their facilities to their financing bodies or managers. In our own experience, the model also proved useful in clarifying the “mechanics” of IPC initiatives to evaluators who were more knowledgeable regarding clinical trials of less complex interventions, such as drugs. For tailoring, the notion of second-order implementation interventions may introduce train-the-trainer concepts into the field as their recipients (implementation support practitioners) are often responsible for tailoring first-order interventions. Additionally, it explores a “ blind spot” in IPC (and possibly other fields), namely regarding who is responsible for such support systems. In addition to hospital management, continuing education formats (such as hospital hygiene days) may have to become more skill-oriented, thus complementing knowledge transfer. Moreover, IPC teams may have to become more multidisciplinary [for instance, by adding a psychologist position (as has been possible at Leipzig University Hospital's Institute of Hygiene, Hospital Epidemiology, and Environmental Medicine)]. Whether IPC-CASCADE will assist implementation science to better conceptualise tailoring, as well as specify its components and effectiveness over and beyond the applied research fields of IPC and antibiotic stewardship, remains to be seen.

## Data Availability

The original contributions presented in the study are included in the article. Further inquiries can be directed to the corresponding author.

## References

[1] Zingg W Storr J Park BJ Ahmad R Tarrant C Castro-Sanchez E 2017 Geneva IPC-think tank. Implementation research for the prevention of antimicrobial resistance and healthcare-associated infections; 2017 Geneva infection prevention and control (IPC)-think tank (part 1). Antimicrob Resist Infect Control. (2019) 8:87. 10.1186/s13756-019-0527-1 31161034 PMC6540528

[2] Gilmartin HM Hessels AJ. Dissemination and implementation science for infection prevention: a primer. Am J Infect Control. (2019) 47(6):688–92. 10.1016/j.ajic.2019.01.023 30850251

[3] Saint S Howell JD Krein SL. Implementation science: how to jump-start infection prevention. Infect Control Hosp Epidemiol. (2010) 31(Suppl 1):S14–7. 10.1086/655991 20929360 PMC3074260

[4] Hancett M. Performance improvement and implementation science: infection prevention competencies for current and future role development. Am J Infect Control. (2012) 40(4):304–8. 10.1016/j.ajic.2012.03.004 22541853

[5] Woods-Hill CZ Papili K Nelson E Lipinski K Shea J Beidas R Harnessing implementation science to optimize harm prevention in critically ill children: a pilot study of bedside nurse CLABSI bundle performance in the pediatric intensive care unit. Am J Infect Control. (2021) 49(3):345–51. 10.1016/j.ajic.2020.08.019 32818579 PMC7889766

[6] Reynolds SS Woltz P Keating E Neff J Elliott J Granger BB. Program evaluation of implementation science outcomes from an intervention to improve compliance with chlorhexidine gluconate bathing: a qualitative study. Dimens Crit Care Nurs. (2022) 41(4):200–8. 10.1097/DCC.0000000000000530 35617584

[7] Tomsic I Heinze NR Chaberny IF Krauth C Schock B von Lengerke T. Implementation interventions in preventing surgical site infections in abdominal surgery: a systematic review. BMC Health Serv Res. (2020) 20(1):236. 10.1186/s12913-020-4995-z 32192505 PMC7083020

[8] Curran GM Bauer M Mittman B Pyne JM Stetler C. Effectiveness-implementation hybrid designs: combining elements of clinical effectiveness and implementation research to enhance public health impact. Med Care. (2012) 50(3):217–26. 10.1097/MLR.0b013e3182408812 22310560 PMC3731143

[9] Bührmann L Driessen P Metz A Burke K Bartley L Varsi C Knowledge and attitudes of implementation support practitioners — findings from a systematic integrative review. PLoS One. (2022) 17(5):e0267533. 10.1371/journal.pone.0267533 35544529 PMC9094539

[10] Baker R Camosso-Stefinovic J Gillies C Shaw EJ Cheater F Flottorp S Tailored interventions to address determinants of practice. Cochrane Database Syst Rev. (2015) 4:CD005470. 10.1002/14651858.CD005470.pub3 PMC7271646 25923419

[11] Kreuter MW Skinner CS. Tailoring: what's In a name? Health Educ Res. (2000) 15:1–4. 10.1093/her/15.1.1 10788196

[12] Ariyo P Zayed B Riese V Anton B Latif A Kilpatrick C Implementation strategies to reduce surgical site infections: a systematic review. Infect Control Hosp Epidemiol. (2019) 40(3):287–300. 10.1017/ice.2018.355 30786946

[13] Marche B Neuwirth M Kugler C Bouillon B Mattner F Otchwemah R. Implementation methods of infection prevention measures in orthopedics and traumatology - a systematic review. Eur J Trauma Emerg Surg. (2021) 47(4):1003–13. 10.1007/s00068-020-01477-z 32914198 PMC8321980

[14] Arbeitskreis Krankenhaus- und Praxishygiene der AWMF (Working Group “Hygiene in Hospital & Practice” of the Association of the Scientific Medical Societies in Germany, AWMF). Händedesinfektion und Händehygiene: AWMF-Register Nr. 029/027, S2k-Leitlinie, Stand 27.08.2016. Hyg Med. (2016) 41(10):254–70. In German

[15] [No authors listed]. Händehygiene in einrichtungen des gesundheitswesens: empfehlung der kommission für krankenhaushygiene und infektionsprävention (KRINKO) beim robert koch-institut (RKI). Bundesgesundheitsblatt Gesundheitsforschung Gesundheitsschutz. (2016) 59(9):1189–220. 10.1007/s00103-016-2416-6. In German 27558147 PMC7079999

[16] Michie S Richardson M Johnston M Abraham C Francis J Hardemann W The behavior change technique taxonomy (v1) of 93 hierarchically clustered techniques: building an international consensus for the reporting of behavior change interventions. Ann Behav Med. (2013) 46(1):81–95. 10.1007/s12160-013-9486-6 23512568

[17] Carey RN Connell LE Johnston M Rothman AJ de Bruin M Kelly MP Behavior change techniques and their mechanisms of action: a synthesis of links described in published intervention literature. Ann Behav Med. (2019) 53(8):693–707. 10.1093/abm/kay078 30304386 PMC6636886

[18] Connell LE Carey RN de Bruin M Rothman AJ Johnston M Kelly MP Links between behavior change techniques and mechanisms of action: an expert consensus study. Ann Behav Med. (2019) 53(8):708–20. 10.1093/abm/kay082 30452535 PMC6636885

[19] von Lengerke T Schock B. Application of psychological theories to organizational behaviour: the case of professional adherence to guidelines in healthcare. In: Körner M Ansmann L Schwarz B Kowalski C, editors. Organizational behaviour in healthcare: Theoretical approaches, methods and empirical results. Zürich: Lit (2018). p. 349–66

[20] von Lengerke T Hartlep I Schipper P Stolz M Tomsic I Krauth C SSI-preventive compliance after tailored interventions for infection prevention and control teams: results of the cluster-randomized controlled WACH-trial in six non-university centres. Antimicrob Resist Infect Control. (2021) 10(Suppl 1):O30. 10.1186/s13756-021-00974-z0

[21] von Lengerke T Lutze B Krauth C Lange K Stahmeyer JT Chaberny IF. Promoting hand hygiene compliance: PSYGIENE—a cluster-randomized controlled trial of tailored interventions. Dtsch Arztebl Int. (2017) 114(3):29–36. 10.3238/arztebl.2017.0029 28179049 PMC5551068

[22] von Lengerke T Ebadi E Schock B Krauth C Lange K Stahmeyer JT Impact of psychologically tailored hand hygiene interventions on nosocomial infections with multidrug-resistant organisms: results of the cluster-randomized controlled trial PSYGIENE. Antimicrob Resist Infect Control. (2019) 8:56. 10.1186/s13756-019-0507-5 30962918 PMC6434638

[23] von Lengerke T Schipper P Hartlep I Stolz M Tomsic I Krauth C Interventionen für Hygieneteams zur Compliance-Förderung: Finale Ergebnisse des WACH-Projekts (vor und während COVID-19). Hyg Med. (2022) 47(Suppl):19–20. Abstract. In German

[24] Robbins SP Judge TA. Essentials of organizational behavior (15th edition). Harlow: Pearson (2021)

[25] Lewin K. Principles of topological psychology. New York: McGraw-Hill (1936)

[26] Michie S van Stralen MM West R. The behaviour change wheel: a new method for characterising and designing behaviour change interventions. Implement Sci. (2011) 6:42. 10.1186/1748-5908-6-42 PMC3096582 21513547

[27] Michie S Atkins L West R. The behaviour change wheel: a guide to designing interventions. London: Silverback (2014).

[28] Cane J O’Connor D Michie S. Validation of the theoretical domains framework for use in behaviour change and implementation research. Implement Sci. (2012) 7(1):37. 10.1186/1748-5908-7-37 22530986 PMC3483008

[29] Atkins L Francis J Islam R O'Connor D Patey A Ivers N A guide to using the theoretical domains framework of behaviour change to investigate implementation problems. Implement Sci. (2017) 12(1):77. 10.1186/s13012-017-0605-9 28637486 PMC5480145

[30] McHugh S Presseau J Luecking CT Powell BJ. Examining the complementarity between the ERIC compilation of implementation strategies and the behaviour change technique taxonomy: a qualitative analysis. Implement Sci. (2022) 17(1):56. 10.1186/s13012-022-01227-2 35986333 PMC9389676

[31] Personnel and organizational requirements for the prevention of nosocomial infections: recommendations from the Commission for Hospital Hygiene and Infection Prevention. Bundesgesundheitsblatt Gesundheitsforschung Gesundheitsschutz. (2009) 52(9):951–62. 10.1007/s00103-009-0929-y. In German 19690813

[32] Empfehlung zum Kapazitätsumfang für die Betreuung von Krankenhäusern und anderen medizinischen Einrichtungen durch Krankenhaushygieniker/innen. Bundesgesundheitsblatt Gesundheitsforschung Gesundheitsschutz. (2016) 59(9):1183–8. 10.1007/s00103-016-2410-z 27558146

[33] Stoliaroff-Pépin A Arvand M Mielke M. [Zur diskussion. Hygienefachpersonal – wann ist der bedarf gedeckt?] in German. Epid Bull. (2018) 45:479–86. 10.17886/EpiBull-2018-054

[34] Knobloch MJ Thomas KV Musuuza J Safdar N. Exploring leadership within a systems approach to reduce health care-associated infections: a scoping review of one work system model. Am J Infect Control. (2019) 47(6):633–7. 10.1016/j.ajic.2018.12.017 30765147

[35] Houghton C Meskell P Delaney H Smalle M Glenton C Booth A Barriers and facilitators to healthcare workers’ adherence with infection prevention and control (IPC) guidelines for respiratory infectious diseases: a rapid qualitative evidence synthesis. Cochrane Database Syst Rev. (2020) 4(4):CD013582. 10.1002/14651858.CD013582 32315451 PMC7173761

[36] von Lengerke T Gohl D Babitsch B. Re-visiting the behavioral model of health care utilization by andersen: a review on theoretical advances and perspectives. In: Janssen C Swart E von Lengerke T, editors. Health care utilization in Germany: theory, methodology, and results. Berlin: Springer (2014). p. 11–28. doi: 10.1007/978-1-4614-9191-0_2

[37] Hale R Powell T Drey NS Gould DJ. Working practices and success of infection prevention and control teams: a scoping study. J Hosp Infect. (2015) 89(2):77–81. 10.1016/j.jhin.2014.10.006 25549828

[38] Knobloch MJ McKinley L Keating J Safdar N. Integrating antibiotic stewardship and infection prevention and control programs using a team science approach. Am J Infect Control. (2021) 49(8):1072–4. 10.1016/j.ajic.2021.01.020 33524453 PMC8060952

[39] Effective Practice and Organisation of Care (EPOC). EPOC Taxonomy; 2015. https://epoc.cochrane.org/epoc-taxonomy. Last access on 03 Nov 2022

[40] Mills T Lawton R Sheard L. Advancing complexity science in healthcare research: the logic of logic models. BMC Med Res Methodol. (2019) 19(1):55. 10.1186/s12874-019-0701-4 30871474 PMC6419426

